# Transitions in frailty states and associated factors: a multistate analysis of the Italian Longitudinal Study on Aging population-based cohort

**DOI:** 10.1016/j.tjfa.2025.100117

**Published:** 2026-01-16

**Authors:** Lucia Galluzzo, Marianna Noale, Stefania Maggi, Marzia Baldereschi, Antonio Di Carlo, Nicola Veronese, Marco Silano, the ILSA Working Group

**Affiliations:** a Department of Cardiovascular, Endocrine-Metabolic diseases, and Ageing, Istituto Superiore di Sanità (ISS), Viale Regina Elena 299, 00161 Roma, Italy; b Neuroscience Institute, Epidemiology of aging and biostatistics group, National Research Council (CNR), Viale G. Colombo 3, 35121 Padova, Italy; c Neuroscience Institute, National Research Council, Via Madonna del Piano 10, Sesto Fiorentino 50019, Italy; d Saint Camillus International University of Health Sciences, Faculty of Medicine, Via Sant’Alessandro 1, 00161 Roma, Italy

**Keywords:** Frailty, Transitions, Cohort study, Aging, Epidemiology

## Abstract

Background: Frailty is recognized as a dynamic and potentially reversible process, but comprehensive studies on its progression/regression are rare.

Objective: To investigate the frequency and characteristics of frailty transitions over time in a representative sample of older Italians.

Design and Participants: As secondary analysis of the Italian Longitudinal Study on Aging (ILSA) population-based cohort, we studied all participants (*n* = 1339; women 47.5 %, age 72.7 ± 5.1) with complete information on changes in frailty status (or death) between consecutive ILSA surveys (T0, T1, T2).

Measurements: Frailty was operationalized according to Fried phenotype, analysing transitions between frailty, or death, during T0-T1, T1-T2 (4-, 5-year length). Transition probability at 1, 3, 5 years was estimated through non-hidden continuous-time Markov models, with death as absorbing state. Factors influencing transitions were evaluated with Cox proportional Hazard Ratios (HR).

Results: We observed 1931 transitions between frailty states and 241 to death. The estimated probability of: maintaining a stable frailty status (∼80 % within 1 year) halved at 5 years; worsening increased steeply over time and was always greater among women; improvement/remission was twice higher at medium (about 20 % among Frail->preFrail women, preFrail->nonFrail men) than short term. Depressive symptoms were the strongest predictor of worsening [nonFrail->Frail: women HR 3.63 (95 %CI 1.45–9.10), men HR 3.78 (95 %CI 2.0–7.13)]. Not having a spouse/partner was associated with a 30 % reduced probability of pre-frailty remission in both sexes.

Conclusions: Our findings confirm the fluctuating nature of frailty with an ample chance of remission/improvement, highlighting the importance of a prompt, multidimensional preventive approach, including psycho-social dimensions.

## Highlights

• 1931 frailty transitions observed during ∼9 years in a population-based cohort ≥65.
• Multistate modelling to predict transitions and related factors at different time intervals.
• Ample chance of frailty remission/improvement for both sexes (∼20 % at 3-year).
• Women have a higher probability of frailty worsening (and resilience) while men of death.
• Socio-psychological factors play a determinant role in the development of frailty.

## 1 Introduction

An unprecedented and increasing number of people are living longer than in the past. However, most of them in late life are forced to face an elevated amount of pathologic conditions and functional limitations that negatively affect personal independence and quality of life [1,2]. A new conceptual framework for older people health and wellbeing has gradually replaced the disease-oriented paradigm of healthy aging, in favour of a multidimensional approach focused also on psychosocial and environmental aspects, and aimed at maximising functional ability reserves as long as possible [3,4]. In this context, frailty – a state of extreme vulnerability to endogenous and exogenous stressors, leading to loss of resilience and increased risk of adverse outcomes – has gained broader recognition over the past decades, and is largely acknowledged as a global public health concern [[5], [6], [7], [8], [9]].

Several definitions and assessment instruments of frailty have been proposed over time [10], until the recent introduction of the complementary construct of intrinsic capacity as measure of functional reserve throughout the life course, based on the interaction between individual physical and mental resources, and the environment [11,12]. The two most common conceptual models of frailty are the physical phenotype [13], which classifies frailty according to five physical components, and the frailty index of cumulative deficits [14,15], based on the proportion of deficit of various nature, including diseases, psychosocial components and other measures of functioning. However, independently of how frailty is defined, some conceptual pillars appear commonly agreed upon. Frailty is recognized as a complex, multidimensional condition, involving different dominions [16], characterized by homeostatic instability in several interrelated systems [5]. It has been also clarified that frailty concept is not synonymous with multimorbidity and disability [6]; with the first it shares biological pathways and an often bidirectional relationship, while the second, together with hospitalization and mortality, represents one of its major outcomes [[17], [18], [19]]. Although common in later life, especially among women, frailty is strongly related to advancing age [[20], [21], [22]] but is not an inevitable and irreversible consequence of the ageing process [23]. On the contrary, it is a dynamic condition, where transitions between frailty levels, including remission, are frequent and may be recurrent [24,25].

According to a well-established hypothesis on the natural history of frailty, its progression should be determined by reduced homeostatic reserve during lifetime [1,5], leading to a series of transitions, in response to episodic or chronic stressors, from states of relative integrity (greater reserve and resilience) to a critical transition point in which the system becomes overwhelmed and cannot restore the necessary physiological integrity, thus failing to return to equilibrium and react to subsequent stressors [26]. In this context, studying how frailty develops and which factors are associated with its progression/regression is crucial to understand the ageing population, identifying individuals at higher risk of becoming more vulnerable, or with higher potential of reaction to a broad range of adverse outcomes, and plan services and intervention to meet their needs. Nonetheless – as also stressed by the inclusion of longitudinal nationally representative surveys among the ten progress indicators of the United Nation Decade of Healthy Ageing 2021–2030 action plan [27] – high-quality, large-scale epidemiological data on progression and natural course of frailty from population-based prospective studies over long time periods are scarce and urgently needed [23,28,29]. Moreover, the few available studies are extremely heterogeneous in the methodological approach and hardly comparable [24], and mainly focused on burden of progression to worst frailty states, rather than on patterns related to improvement or remission [25,30].

As part of a systematic comprehensive study of frailty conducted in a large population-based cohort of older Italians [19], this study aimed (1) to examine the changes in frailty status observed between three longitudinal waves covering a period of about 9 years, (2) to estimate the transition probability at short, medium and long term using multistate modelling, with death as absorbing state, and (3) to analyse factors influencing the transitions, with a special focus on sex differences and regression probabilities.

## 2 Methods

### 2.1 Study design and sample

The present analysis is part of the Italian Longitudinal Study on Aging (ILSA), an extensive epidemiological study aimed at investigating frequency, risk and protective factors of age-associated conditions, including physical and functional changes, in a population-based cohort of older Italians. The study was conducted according to the guidelines laid down in the World Medical Association Declaration of Helsinki. All procedures were approved by the Bioethics Committees of the participating centres and written informed consent was obtained before starting the study.

The ILSA protocol was described in details elsewhere [31]. In brief, a sample of 5632 subjects aged 65–84 years was randomly extracted from the demographic lists of eight municipalities located across Italy, stratifying by sex assigned at birth and 5-year age classes, using an equal allocation strategy. The ILSA cohort was first examined in 1992–1993 (T0), and extensively re-examined in two longitudinal waves carried out in 1995–1996 (T1) and 2000–2001 (T2) with the same two-phase design. In phase 1 (screening), all participants underwent: (a) a personal interview on socio-demographic characteristics, family and medical history, self-reported health problems and risk factors; (b) a nurse visit, including a fasting blood sample; (c) a physical examination by a physician, comprising clinical and functional assessment, neurological examination, neuropsychological battery, and diagnostic tests. In phase 2 (clinical confirmation), participants who screened positive for one of the chronic conditions under study were examined by a specialist to confirm or exclude suspected diagnoses, on the basis of clinical examination and the review of medical records, according to standardized criteria [32]. Detailed information on vital status and cause-specific mortality have been periodically collected through record-linkage with the national mortality register.

The frailty study subset included a total of 2239 participants with adequate information for retrospective assessment of frailty in one or more ILSA surveys (T0 *n* = 1992; T1 *n* = 1279; T2 *n* = 1094).

### 2.2 Frailty assessment and measurement of transitions

The procedures adopted to operationalize frailty status from ILSA dataset is fully described in a previous article [19]. Frailty was defined according to an adaptation of the physical phenotype criteria [13]: a) unintentional weight loss >5 kg in the past year; b) exhaustion measured by item 21 and the overall score of the Geriatric Depression Scale (GDS-30) [33]; c) weakness defined as inability to perform the chair-stand test without help [34]; d) slowness based on a mean ≥7 s on two measurements of the 5-meter gait speed test [34]; e) low physical activity or sedentary lifestyle based on a retrospective questionnaire administered at T2, combined with selected items of the Activities of Daily Living (ADL) [35] and Instrumental Activities of Daily Living (IADL) [36] scales. Subjects with reliable information on all criteria were classified as frail if they met at least 3 out of 5 criteria, pre-frail in case of 1 or 2 of them, and as non-frail if they met none (0=non-frail; 1–2=pre-frail; ≥3=frail).

Only changes in frailty status occurred between consecutive assessments (T0-T1, T1-T2) were included in the present analysis, classifying attrition other than death as ‘missing’. Frailty transitions were analysed also in terms of worsening (from non-frail to pre-frail, or frail; from pre-frail to frail), improvement (from pre-frail to non-frail; from frail to pre-frail, or non-frail) and stability (no changes).

### 2.3 Covariates

The following socio-demographic and health conditions, often reported as associated to frailty, were examined in relation to frailty status and as potential predictors of transitions over time.

*Socio-demographic conditions*. Sex; age; education (years of schooling 0–3 or 4–7 vs. ≥8); marital status (non-married vs. married or living with a partner); cohabitation status (living alone vs. living with someone).

*Lifestyle habits and nutrition.* Smoking status (current smoker or ex-smoker vs. never smoker); alcohol consumption (current drinker or ex-drinker vs. abstainers); BMI (kg/m^2^) categorized into underweight or obesity (<18.5 or ≥30) vs. normal weight or overweight (18.5–24.9 or 25–29.9); adherence to the Mediterranean diet according to a 10-point scale (0–3 low adherence, 4–5 moderate, 6–9 high) [37], based on the ILSA 49-item semi-quantitative food frequency questionnaire.

*Age-related diseases and functional impairments*. Cognitive impairment according to Mini-Mental State Examination (MMSE) [38] (mild/severe MMSE score <24 vs. absent ≥24); depressive symptoms as assessed through GDS-30 [33] (mild/severe GDS score ≥10 vs. absent <10); clinical diagnosis of hypertension, myocardial infarction, angina pectoris, cardiac arrhythmia, congestive heart failure, diabetes, peripheral artery disease, dementia, parkinsonism, stroke, and distal symmetric neuropathy [32]; comorbidity (diagnosis of ≥2 pathologic conditions); self-reported falls (≥1 during past 12 months).

### 2.4 Statistical analysis

The characteristics of participants by frailty conditions at entry in the two time periods of observation were summarized as mean ± standard deviation (SD) for quantitative variables, and as counts and percentages for categorical ones. For continuous variables, normal distributions were tested using the Kolmogorov-Smirnov test. Comparisons between groups were evaluated using the Chi-square or Fisher exact test for categorical variables, and generalized linear models, testing for homoscedasticity through Levene’s test, or the Wilcoxon sum rank test for quantitative variables. The frequencies of the transitions among the three frailty states and death were calculated for the whole sample and by sex for the two follow-up intervals T0-T1 and T1-T2 (mean duration 4 and 5 years, respectively).

Transition intensities and probabilities between frailty states were estimated considering non-hidden continuous-time Markov models, with death as an absorbing state (SupplementaryFigure 1, SupplementaryFigure 2). The aims of the multistate analysis were: (1) to estimate transition intensity matrix parameters for each time interval (T0-T1; T1-T2) by maximizing the likelihood function based on the observed frailty states at each assessment; (2) to estimate the transition probability matrix at short (1 year), medium (3 years) and long term (5 years). To investigate factors associated with transitions, proportional intensity models (Cox-type) were applied, considering stability in frailty status as the reference. Covariates were selected considering significant association with frailty status at T0 and T1 in bivariate analyses, and included both time-constant variables (sex, education) and time-varying variables. Results are expressed in terms of adjusted Hazard Ratios (HR) and 95 % Confidence Intervals (CI) for each covariate. Model convergence was optimized applying a quasi-Newton optimization algorithm (BFGS). Missing data were censored and considered missing completely at random; analyses were repeated stratifying by gender. The estimated survival probability (in years from each frailty status assessment to death) were plotted from the transition probability matrices, adjusting for age and sex.

All tests were two-sided with a significance level of *p* < 0.05. The analyses were conducted using SAS (version 9.4) and the *msm* package in R statistical software.

## 3 Results

### 3.1 Descriptive analysis of transitions

Out of the 2239 older individuals included in the ILSA frailty study (prevalence 4.0 % frailty, 44.6 % pre-frailty; incidence rate per 1000 person-years 7.3 frailty, 83.7 pre-frailty [19]), transitions in frailty states were detected in 1372 subjects with complete information in at least two frailty assessments.

**Fig. 1:**
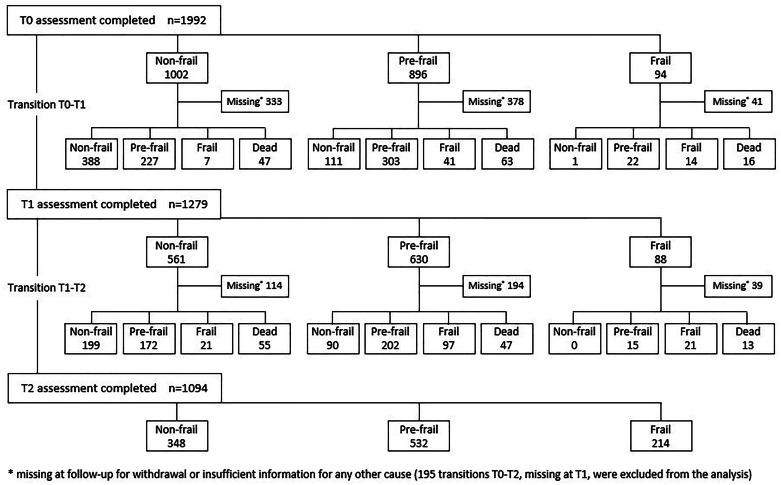
Flowchart of the study of frailty transitions in the ILSA cohort (n).

Transitions in both T0-T1 and T1-T2 were observed in 754 individuals with available information at all three time points, while 360 subjects had transitions only for the period T0-T1, 63 for T1-T2 and 195 for T0-T2 because of missing frailty assessment at T2, T0 and T1, respectively. After the exclusion of transitions between non-consecutive assessments (*n* = 195), the final sample was composed of 1339 persons, some of whom experienced more than one transition to frailty or death over time (women 47.5 %; mean age 72.7 ± 5.1), for a total of 1931 changes between frailty states (T0-T1 *n* = 1114; T1-T2 *n* = 817) and 241 transitions to death observed during the overall follow-up period (mean 9 years). The flow-chart in Fig. 1 represents the various stages of the study of transitions, quantifying the number of subjects involved in each variation in frailty status over time.

The characteristics of the study sample by frailty status at entry in each follow-up segments are illustrated in Table 1. At both starting points, pre-frail and frail subjects were older, more likely to be female, less educated, less frequently married, had a lower proportion of current drinkers or smokers and were more likely to be in the extreme categories of BMI (underweight or obese). They appeared in overall worst health conditions, as shown by the higher prevalence of cognitive impairment and depressive symptoms (more than four times for frail than non-frail), comorbidity and falls (nearly double for frail individuals), and all investigated neurological diseases. With the exception of angina and congestive heart failure, and arrhythmia at T1, all other cardiovascular conditions showed no significant difference in the distribution by frailty status. Although not reaching statistical significance, frail subjects had lower levels of adherence to the Mediterranean diet.

**Table 1:** Characteristics of participants by frailty status at entry in the two follow-up intervals of the ILSA frailty transitions study.

	T0				T1			
	Non-frail	Pre-frail	Frail	p-value	Non-frail	Pre-frail	Frail	p-value
	(*n* = 1002)	(*n* = 896)	(*n* = 94)		(*n* = 561)	(*n* = 630)	(*n* = 88)	
Sex**,** female	391 (39.0)	551 (61.5)	64 (68.1)	<0.0001	201 (35.8)	365 (57.9)	55 (62.5)	<0.0001
Age, years	72.0 ± 5.0	73.8 ± 5.2	77.1 ± 4.9	<0.0001	74.9 ± 4.7	76.1 ± 5.1	78.9 ± 5.6	<0.0001
Years of schooling				<0.0001				<0.0001
0–3	228 (23.7)	261 (30.1)	41 (44.6)		111 (20.6)	189 (31.1)	35 (41.7)	
4–7	436 (45.3)	351 (40.5)	28 (30.4)		258 (47.9)	243 (40.0)	31 (36.9)	
≥8	299 (31.0)	255 (29.4)	23 (25.0)		170 (31.5)	176 (28.9)	18 (21.4)	
Marital status, married	709 (70.8)	502 (56.0)	53 (56.4)	<0.0001	379 (67.6)	342 (54.3)	42 (47.7)	<0.0001
Living arrangements, alone	160 (16.0)	189 (21.1)	13 (13.8)	0.0083	103 (18.4)	135 (21.5)	12 (13.8)	0.1429
Smoking status				<0.0001				0.1059
current smoker	196 (19.6)	131 (14.6)	7 (7.4)		78 (13.9)	71 (11.2)	9 (10.2)	
ex-smoker	383 (38.2)	252 (28.1)	23 (24.5)		69 (12.3)	54 (8.6)	10 (11.4)	
never smoker	423 (42.2)	513 (57.3)	64 (68.1)		414 (73.8)	505 (80.2)	69 (78.4)	
Alcohol consumption				0.0299				<0.0001
current drinker	750 (74.8)	641 (71.5)	57 (60.6)		424 (75.6)	411 (65.2)	50 (56.8)	
ex-drinker	115 (11.5)	113 (12.6)	19 (20.2)		24 (4.3)	29 (4.6)	8 (9.1)	
never drinker	137 (13.7)	141 (15.9)	18 (19.2)		113 (20.1)	190 (30.2)	30 (34.1)	
BMI kg/m^2^				0.0323				<0.0001
underweight or obesity	195 (19.7)	216 (24.5)	22 (25.0)		102 (18.8)	146 (25.2)	26 (41.3)	
normal weight or overweight	797 (80.3)	665 (75.5)	66 (75.0)		441 (81.2)	433 (74.8)	37 (58.7)	
Mediterranean Diet Score				0.1520				0.2872
low adherence	116 (11.6)	111 (12.4)	11 (11.7)		112 (20.0)	146 (23.1)	25 (28.7)	
moderate adherence	386 (38.5)	359 (40.1)	48 (51.1)		271 (48.3)	289 (45.9)	41 (47.2)	
high adherence	500 (49.9)	426 (47.5)	35 (37.2)		178 (31.7)	195 (31.0)	21 (24.1)	
Cognitive impairment, MMSE <24	69 (6.9)	99 (11.1)	26 (27.7)	<0.0001	31 (5.6)	94 (15.1)	25 (31.7)	<0.0001
Depressive symptoms, GDS ≥10	215 (21.8)	372 (42.3)	79 (85.0)	<0.0001	86 (15.3)	246 (39.1)	79 (89.8)	<0.0001
Hypertension	640 (72.2)	580 (71.9)	64 (74.4)	0.8820	376 (77.5)	439 (80.4)	63 (78.8)	0.5255
Myocardial Infarction	62 (6.2)	77 (8.6)	10 (10.8)	0.0640	44 (7.9)	50 (92.0)	77 (87.5)	0.3245
Angina	64 (6.4)	76 (8.5)	12 (12.8)	0.0372	44 (7.9)	88 (14.0)	12 (13.6)	0.0031
Arrhythmia	242 (24.6)	234 (26.2)	30 (32.6)	0.2130	163 (29.8)	177 (28.6)	39 (44.8)	0.0079
Heart Failure	21 (2.1)	49 (5.5)	16 (17.0)	<0.0001	14 (2.5)	30 (4.8)	16 (18.2)	<0.0001
Diabetes	106 (10.6)	123 (13.8)	14 (14.9)	0.0776	55 (9.9)	67 (10.8)	14 (15.9)	0.2411
Arteriopathy	43 (5.0)	54 (7.3)	6 (8.3)	0.1272	28 (31.8)	32 (26.2)	3 (13.6)	0.2173
Dementia	3 (0.3)	12 (1.3)	13 (14.1)	<0.0001	0 (0.0)	17 (2.8)	14 (16.9)	<0.0001
Parkinsonism	9 (0.9)	14 (1.6)	10 (12.1)	<0.0001	4 (0.7)	22 (3.6)	11 (15.7)	<0.0001
Stroke	32 (3.7)	36 (4.7)	15 (20.6)	<0.0001	16 (3.3)	24 (4.6)	11 (18.6)	<0.0001
Neuropathy	50 (5.1)	61 (7.1)	12 (15.0)	0.0013	30 (5.7)	53 (9.3)	15 (21.4)	<0.0001
Comorbidity, ≥2 diseases	343 (34.2)	375 (41.9)	59 (62.8)	<0.0001	223 (39.8)	294 (46.7)	58 (65.9)	<0.0001
Falls, ≥1 past year	148 (14.8)	166 (18.5)	25 (26.6)	0.0038	191 (34.1)	267 (42.4)	55 (62.5)	<0.0001
Frailty phenotype criteria								
weight loss	0 (0.0)	106 (11.8)	32 (34.0)	<0.0001	0 (0.0)	43 (6.8)	18 (20.5)	<0.0001
exhaustion	0 (0.0)	191 (21.3)	72 (76.6)	<0.0001	0 (0.0)	129 (20.5)	73 (83.0)	<0.0001
weakness	0 (0.0)	71 (7.9)	64 (68.1)	<0.0001	0 (0.0)	26 (4.1)	58 (65.9)	<0.0001
slowness	0 (0.0)	626 (69.9)	91 (96.8)	<0.0001	0 (0.0)	503 (79.8)	88 (100.0)	<0.0001
low physical activity	0 (0.0)	132 (14.7)	51 (54.3)	<0.0001	0 (0.0)	101 (16.0)	52 (59.1)	<0.0001

BMI, Body Mass Index; MMSE, Mini Mental State Examination; GDS, Geriatric Depression Scale.

Data are expressed as number (percentage) for categorical variables and as mean ± standard deviation for continuous variables; percentages may not total 100 because of rounding, or missing information.

Table 2 provides the counts and rates of transitions between the three frailty states or death in each of the two follow-up intervals. As shown also in SupplementaryTable 1, about half of participants remained in the same frailty level over time and stability was more frequent for non-frailty and pre-frailty conditions. In both observation periods, overall worsening transitions were more common among women and mainly concentrated in changes from fit to pre-frail; men tended to transition more frequently to death, with a rate from frailty to death almost double than women (T0-T1 46.7 % men vs. 23.7 % women; T1-T2 33.3 % vs. 21.4 %). About 10 % of participants experienced at least one change towards a better frailty state, which were mostly based on pre-frailty remission and covered almost all improvements in frailty condition among men. Finally, the occurrence of direct transitions from robust to frail, and vice versa, were quite rare.

**Table 2:** Transitions between frailty states, or death, in the ILSA cohort during each follow-up interval, by sex.

		*To*							
		Non-frail		Pre-frail		Frail		Dead	
		n	%	n	%	n	%	n	%
**T0-T1**									
Women									
*From*	Non-frail	123	50.0	108	43.9	2	0.8	13	5.3
	Pre-frail	54	17.7	194	63.6	26	8.5	31	10.2
	Frail	1	2.6	17	44.7	11	28.9	9	23.7
Men									
*From*	Non-frail	265	62.6	119	28.1	5	1.2	34	8
	Pre-frail	57	26.8	109	51.2	15	7	32	15
	Frail	0	0	5	33.3	3	20.0	7	46.7
Whole									
*From*	Non-frail	388	58.0	227	33.9	7	1.0	47	7.0
	Pre-frail	111	21.4	303	58.5	41	7.9	63	12.2
	Frail	1	1.9	22	41.5	14	26.4	16	30.2
**T1-T2**									
Women									
*From*	Non-frail	49	33.8	67	46.2	10	6.9	19	13.1
	Pre-frail	35	14.6	123	51.5	58	24.3	23	9.6
	Frail	0	0	9	32.1	13	46.4	6	21.4
Men									
*From*	Non-frail	150	49.7	105	34.8	11	3.6	36	11.9
	Pre-frail	55	27.9	79	40.1	39	19.8	24	12.2
	Frail	0	0	6	28.6	8	38.1	7	33.3
Whole									
*From*	Non-frail	199	44.5	172	38.5	21	4.7	55	12.3
	Pre-frail	90	20.6	202	46.3	97	22.2	47	10.8
	Frail	0	0.0	15	30.6	21	42.9	13	26.5

Transition rates (%) calculated on the total number of participants with data on frailty or death at both time points delimiting the intervals.

### 3.2 Estimated frailty transition probability

Table 3 shows the transition probabilities among frailty states estimated through multistate modelling for different time intervals indicative of short, medium and long term. The chance of remaining in the same frailty state decreased steeply over time and was mainly concentrated on pre-frailty for females (83.4 %, 63.1 %, 51.7 % at 1- 3-, 5-year) and non-frailty for males (83.6 %, 61.7 %, 48.4 % respectively). The probability of any change in frailty status, either improved or worsened, tended to be more than double at medium compared to short term, remaining nearly unchanged or with smaller increases at long term. The transition probabilities towards improved frailty levels were mostly based on changes from frailty to pre-frailty among women and from pre-frailty to non-frailty in men (both ∼20 % at 3-year). The chance of transitioning to a worst frailty state was always higher among women, and was mainly attributable to changes from fit to pre-frail in both sexes. A non-frail woman had a 0.9 % probability of becoming frail at short term, 5.1 % at medium and 9.1 % at long term vs. a corresponding chance of 0.6 %, 3.2 % and 5.8 % for a man.

**Table 3:** Estimated transition probabilities (95 % Confidence Intervals) between frailty states or death, at short, medium and long term, by sex.

		1-year					3-year					5-year			
		*To*					*To*					*To*			
		Non-frail	Pre-frail	Frail	Dead		Non-frail	Pre-frail	Frail	Dead		Non-frail	Pre-frail	Frail	Dead
Women															
*From*	Non-frail	78.6	18.6	0.9	1.9		51.5	37.3	5.1	6.1		36.4	43.2	9.1	11.3
		(78.1–78.5)	(18.1–20.0)	(0.8–0.10)	(0.9–2.5)		(51.0–53.6)	(34.6–38.4)	(4.7–5.1)	(5.4–7.0)		(37.3–38.0)	(40.9–44.2)	(8.9–9.7)	(11.5–12.2)
	Pre-frail	6.5	83.4	7.9	2.2		13.0	63.1	15.2	8.7		15.1	51.7	17.0	16.2
		(6.3–6.6)	(81.9–83.8)	(7.7–8.0)	(2.0–4.1)		(11.0–14.8)	(64.7–66.6)	(14.8–15.7)	(5.7–9.7)		(13.6–17.2)	(49.5–54.8)	(16.3–18.0)	(13.5–17.0)
	Frail	0.4	10.9	75.0	13.7		2.4	21.0	44.2	32.4		4.4	23.4	27.9	44.3
		(0.3–0.5)	(9.9–12.2)	(75.9–77.1)	(10.1–13.9)		(2.3–2.5)	(18.9–24)	(42.1–45.1)	(31.6–35.5)		(4.4–5.6)	(25.1–26.8)	(23.5–28.9)	(42.3–45.3)
Men															
*From*	Non-frail	83.6	13.3	0.6	2.5		61.7	26.9	3.2	8.2		48.4	31.3	5.8	14.5
		(81.9–84.4)	(12.3–14.8)	(0.4–0.7)	(1.7–3.2)		(59.0–64.1)	(24.3–29.8)	(2.6–3.6)	(6.8–9.4)		(45.9–51.4)	(29.5–33.5)	(5.1–7.2)	(13.1–15.3)
	Pre-frail	10.0	79.8	6.7	3.5		20.3	55.0	12.6	12.1		23.6	41.5	13.5	21.4
		(9.5–11.8)	(78.4–80.9)	(5.0–7.7)	(2.7–4.7)		(17.1–23.4)	(51.5–58.9)	(11.0–14.9)	(8.9–14.7)		(21.7–25.0)	(39.0–42.2)	(12.5–14.9)	(20.1–23.5)
	Frail	0.4	5.6	76.3	17.7		2.0	10.4	45.4	42.2		3.7	11.2	27.7	57.4
		(0.1–0.6)	(1.4–9.0)	(70.5–80.6)	(14.4–22.6)		(1.5–2.6)	(8.5–14.9)	(39.9–48.9)	(36.2–47.1)		(2.8–5.3)	(8.8–15.6)	(21.3–36.7)	(48.2–63.5)
Whole															
*From*	Non-frail	82.2	14.7	0.7	2.4		58.4	30.1	3.9	7.6		44.0	35.6	7.0	13.4
		(81.1–82.9)	(3.8–15.9)	(0.6–0.8)	(0.2–3.1)		(56–60.6)	(28.8–32.6)	(3.6–4.3)	(6.6–7.8)		(41.8–45.6)	(34.3–37.2)	(6.3–7.7)	(12.2–15.3)
	Pre-frail	7.7	82.2	7.4	2.7		15.9	59.9	14.1	10.1		18.7	47.4	15.5	18.4
		(7.1–8.4)	(81.0–83.0)	(6.7–8.3)	(1.7–3.2)		(14.4–16.9)	(57.7–61)	(12.5–15.4)	(8.7–12.2)		(18.2–19.9)	(45.6–49.3)	(13.8–15.9)	(17.4–20.1)
	Frail	0.4	8.7	75.6	15.3		2.4	16.6	44.6	36.4		4.4	18.2	27.8	49.6
		(0.3–0.6)	(6.8–12.1)	(71.7–78.8)	(13.9–17.7)		(1.8–3.2)	(13.1–21.3)	(40.1–50.2)	(31.1–42.4)		(3.7–6.1)	(15.8–23.5)	(21.3–31)	(43.6–54.6)

### 3.4 Estimated survival probability for each frailty state

Frail subjects had the highest estimated probability of transitioning to death, followed by pre-frail and non-frail (Table 3). The chance of dying, as expected, increased with age and was considerably greater among frail men than women (42.2 % vs. 32.4 % at 3 years). As shown in the survival plots presented as SupplementaryFigure 3, the estimated survival probability of frail individuals diminished quite rapidly within the first years after baseline, in particular for men. The gap between the greater survival probability for non-frail than pre-frail individuals widened over time among men, while tended to flatten out for women.

### 3.5 Factors influencing transitions in frailty states

Table 4 shows the sex-specific transition intensities expressed for each covariate (in bold those reaching statistical significance); sex-aggregated findings are illustrated in SupplementaryTable 2. The presence of depressive symptoms was by far the strongest predictor of worsening transitions, with hazard ratios indicating a four times higher probability of transition from fit to frail and a 40 % increased risk of passing from pre-frailty to frailty; these findings were essentially overlapping for men and women. The coexistence of two or more chronic conditions was associated with a greater risk of developing frailty from pre-frailty, especially for women (increased risk 60 % women vs. 40 % men).

**Table 4:** Factors influencing transitions from frailty states according to the multistate model adjusting for all covariates, by sex.

Covariates	Transitions								
	Non-frail to			Pre-frail to			Frail to		
	Pre-frail	Frail	Dead	Non-frail	Frail	Dead	Non-frail	Pre-frail	Dead
*Women*									
Age, years	1.00 (0.97–1.04)	1.06 (0.98–1.16)	**1.07** **(1.02–1.12)**	**0.92** **(0.87–0.96)**	**1.08** **(1.03–1.12)**	**1.08** **(1.03–1.13)**	1.31 (0.68–2.49)	0.99 (0.90–1.08)	1.13 (0.89–1.43)
Schooling, <8 years	1.09 (0.76–1.58)	0.87 (0.27–2.82)	0.84 (0.44–1.62)	1.26 (0.76–2.09)	0.83 (0.45–1.54)	0.92 (0.52–1.61)	0.02 (0–18,291.59)	1.05 (0.31–3.55)	1.76 (0.16–19.5)
Marital status, not married	1.21 (0.82–1.78)	0.65 (0.23–1.88)	0.89 (0.43–1.86)	**0.68** **(0.39–0.98)**	0.99 (0.59–1.64)	1.44 (0.84–2.49)	0.33 (0–291.03)	1.02 (0.37–2.82)	0.92 (0.09–9.9)
Liv. arrangement, living alone	0.85 (0.56–1.29)	0.85 (0.23–3.18)	1.73 (0.83–3.59)	1.27 (0.67–2.39)	0.9 (0.53–1.54)	1.12 (0.69–1.82)	–	1.23 (0.36–4.22)	0.26 (0.01–5.84)
Smoking habit, smoker/ex	1.08 (0.77–1.51)	1.06 (0.41–2.76)	0.85 (0.48–1.50)	1.1 (0.67–1.78)	0.61 (0.40–1.04)	**1.88** **(1.09–3.24)**	–	0.47 (0.20–1.12)	4.46 (0.3–67.49)
Alcohol habit, drinker/ex	0.84 (0.55–1.27)	0.59 (0.13–2.69)	1.86 (0.97–3.57)	1.3 (0.77–2.19)	0.66 (0.30–1.45)	0.66 (0.30–1.45)	**1.96** **(1.17–3.29)**	0 (0–213.53)	0.66 (0.17–2.63)
BMI, underweight /obese	1.12 (0.8–1.57)	1.09 (0.42–2.87)	0.96 (0.51–1.80)	0.96 (0.51–1.80)	0.73 (0.42–1.24)	**1.56** **(1.01–2.41)**	1.08 (0.67–1.76)	0 (0–482.66)	1.42 (0.55–3.66)
Comorbidity, ≥2	1.00 (0.71–1.41)	1.93 (0.79–4.73)	1.53 (0.88–2.67)	0.66 (0.41–1.08)	**1.62** **(1.06–2.45)**	**2.84** **(1.81–4.46)**	0.04 (0–67.6)	0.66 (0.29–1.54)	3.18 (0.44–23.24)
Cognitive impairment, MMSE <24	1.13 (0.64–2.00)	0.61 (0.13–2.85)	0.9 (0.33–2.40)	0.97 (0.44–2.12)	1.42 (0.83–2.42)	1.15 (0.60–2.18)	–	0.61 (0.21–1.82)	1.35 (0.07–27.82)
Depressive symptoms, GDS ≥10	1.13 (0.81–1.59)	**3.63** **(1.45–9.1)**	1.07 (0.59–1.96)	**0.70** **(0.44–0.99)**	**1.43** **(1.04–2.18)**	1.2 (0.79–1.84)	–	0.68 (0.24–1.93)	1.5 (0.11–19.6)
Falls, ≥1	0.83 (0.58–1.19)	1.52 (0.60–3.88)	0.90 (0.49–1.64)	1.11 (0.7–1.77)	1.36 (0.88–2.09)	0.61 (0.37–1.05)	–	0.80 (0.32–1.97)	0.59 (0.08–4.17)
*Men*									
Age, years	1.01 (0.99–1.03)	**1.08** **(1.02–1.14)**	**1.09** **(1.06–1.12)**	**0.95** **(0.92–0.98)**	**1.08** **(1.04–1.12)**	**1.06** **(1.03–1.1)**	1.00 (0.70–1.43)	0.99 (0.92–1.06)	**1.11** **(1.00–1.23)**
Schooling, <8 years	0.97 (0.78–1.21)	0.95 (0.47–1.95)	0.95 (0.67–1.34)	0.92 (0.67–1.27)	1.1 (0.72–1.70)	1 (0.69–1.43)	5.18 (0–209,705.2)	1.24 (0.46–3.36)	0.49 (0.14–1.67)
Marital status, not married	1.1 (0.83–1.46)	1.18 (0.56–2.46)	0.97 (0.60–1.58)	**0.66** **(0.45–0.98)**	0.89 (0.61–1.31)	1.36 (0.94–1.97)	2.14 (0.09–53.89)	1.21 (0.53–2.78)	0.65 (0.19–2.23)
Liv. arrangement, living alone	0.95 (0.67–1.35)	0.4 (0.13–1.25)	1.53 (0.89–2.64)	1.12 (0.68–1.86)	0.9 (0.55–1.45)	1.36 (0.90–2.04)	0.14 (0–452,691.8)	0.88 (0.27–2.88)	0.48 (0.05–4.91)
Smoking status, smoker/ex	1.01 (0.77–1.32)	0.93 (0.45–1.94)	1.1 (0.70–1.73)	1.25 (0.84–1.86)	0.7 (0.48–1.02)	**1.61** **(1.03–2.51)**	0.15 (0.01–4.06)	0.49 (0.24–1.01)	1.39 (0.44–4.35)
Alcohol habit, drinker/ex	0.93 (0.75–1.15)	0.63 (0.30–1.29)	**1.44** **(1.03–2.01)**	1.14 (0.85–1.55)	0.81 (0.54–1.21)	0.81 (0.54–1.21)	**1.84** **(1.33–2.55)**	0.03 (0–132)	0.83 (0.34–2.01)
BMI, underweight /obese	1.12 (0.88–1.43)	1.38 (0.69–2.78)	1.11 (0.74–1.66)	1.11 (0.74–1.66)	0.69 (0.48–1.01)	**1.62** **(1.14–2.29)**	1.04 (0.72–1.50)	0.05 (0–1215.22)	1.71 (0.78–3.77)
Comorbidity, ≥2	1.09 (0.89–1.34)	1.39 (0.76–2.54)	0.94 (0.68–1.31)	0.57 (0.42–0.78)	**1.37** **(0.99–1.89)**	**1.98** **(1.45–2.69)**	0.32 (0.01–7.25)	1.04 (0.52–2.09)	1.30 (0.53–3.19)
Cognitive impairment, MMSE <24	1.24 (0.86–1.8)	0.94 (0.33–2.69)	1.09 (0.60–1.98)	1.15 (0.70–1.89)	1.38 (0.90–2.09)	1.1 (0.70–1.73)	0.06 (0–2167.61)	0.5 (0.18–1.4)	1.41 (0.48–4.14)
Depressive symptoms, GDS ≥10	1.12 (0.88–1.44)	**3.78** **(2.00–7.13)**	1.27 (0.86–1.87)	**0.66** **(0.47–0.92)**	**1.44** **(1.03–2.00)**	1.24 (0.90–1.7)	2.13 (0–101,868.6)	0.64 (0.25–1.62)	3.45 (0.34–34.78)
Falls, ≥ 1	0.91 (0.71–1.16)	1.43 (0.74–2.76)	0.63 (0.4–0.97)	1.04 (0.74–1.44)	1.29 (0.92–1.81)	0.77 (0.54–1.11)	0.05 (0–1942.92)	0.88 (0.41–1.88)	1.02 (0.37–2.82)

Values are expressed as Hazard Ratios (95 % Confidence Intervals), using as reference the complementary category/ies at lower frailty risk from bivariate analysis.

Statistically significant association (CI not including 1.00) are presented in bold.

No significant association was detected for changes from frailty to pre-frailty, while a significant inverse association with pre-frailty remission was observed for older age, depressive symptoms, being unmarried or not having a partner. As suggested also by bivariate analysis, drinkers or ex-drinkers, in comparison to abstainers, showed a 90 % increased probability of total remission from frailty. An unexpected result is the significant association between falls and an almost 40 % reduced probability of death among non-frail men.

## 4 Discussion

In response to the need for a better knowledge on how frailty develops over time, we explored changes in frailty status in a nationally representative cohort of older individuals extensively investigated over a period of about 9 years, with an ongoing periodic follow-up of mortality. Data derived from the descriptive analysis of transitions were used to feed multistate models to describe how individuals moved between frailty states at 1-, 3- and 5-year intervals and to identify factors influencing the different transitions.

The added value of the present study stands on its use of a large, population-based dataset with a long period of observation, which allowed the application of multistate modelling to predict transitions between frailty states at different time intervals. A similar methodology was recently applied to primary care data, based on a routinely collected electronic frailty index [39], and in a study nested within a prevention trial, with shorter periods of observation on a smaller sample [40]. The use of multistate models amplifies the magnitude of findings from the descriptive analysis of transitions that otherwise should be interpreted with caution, being based on simple frequencies measured at arbitrary intervals, which not always reflect the actual underlying progression of frailty status, since states between each assessment remain unknown.

Our findings of a sharply decreasing probability of maintaining a stable frailty condition after one year – about 80 % for all frailty states – accompanied by a chance of frailty changes almost double at three years, strongly recommend the need for planning interventions at short/medium term. This is in agreement with Walsh et al. finding of a progressive reduction of the mean time spent in each frailty category, with the longest period spent in severe frailty at all ages [39].

The present analysis showed various differences in the progression of frailty between men and women, confirming the well-established relationship between frailty and female sex, also demonstrated by the significantly higher frequency observed among women in this same cohort [19]. Consistently with other studies, we found that women were more likely to experience worsening transitions [[40], [41], [42]]. Furthermore, although the chance of improvement in our analysis was nearly the same for both sexes, it was mostly attributable to recovering from frailty to pre-frailty in women, and from pre-frailty to non-frailty in men. Similarly, frailty stability over time was mainly concentrated on pre-frail women and non-frail men. This is in accordance with an extensive meta-analysis of transitions among community-dwelling older people, reporting that among robust participants, men were more likely to remain robust and women to transition to pre-frail [24]. Other interesting differences according to sex resulted from the survival analysis, which confirmed a lower survival probability among men and in general among frail subjects [[42], [43], [44], [45]]. In addition, it also provided evidence for a marked decrease in the survival probability of pre-frail and, above all, frail men, in contrast with a reduced trend in women where the differential survival by frailty status was not so evident. These gender-specific features may be attributable to the so-called male–female health–survival paradox (i.e. women tend to have poorer health conditions but greater longevity) [46]. Our findings support the hypothesis of a parallel sex-frailty paradox [47], suggesting that women in comparison to men of the same age are more likely to be frail (higher prevalence) and to have a greater frailty burden (more frequent worsening transitions), but are more resilient (higher survival probability and progressive reduction of the differential survival by frailty states). This hypothesis warrants further investigation to identify biological, environmental and social trajectories over time.

No particular sex differences were present in regards to factors influencing frailty transitions. Remarkably, two of the main predictors identified both belonged to the psychological and relational domain: depressive symptoms (linked to a higher probability of changes from fit to frail and a parallel negative association with pre-frailty remission) and not having a spouse/partner (responsible for a significant reduced chance of pre-frailty regression). The strong and often bidirectional relationship between frailty and depression is well-established [[48], [49], [50]] also in association with frailty transitions [40,51]. As regards marital status, already reported as associated with incident frailty in a similar Italian cohort [52], it must be noticed that according to our findings (Table 4), the influence on frailty remission was exerted only by being married or living with a partner, which implies the existence of a long lasting affective relationship, while was not significant for living with someone else. These results are consistent with previous literature underlining the relation between frailty and reduced interpersonal engagement [53], emphasizing the importance of a person-centred, multidimensional, integrated approach to frailty, including also social and psychological aspects, and going beyond the disease oriented vision of older age [4,54,55].

The main limitations of the present study are due to the approach used for frailty assessment, using data originally collected with different objectives. This might have led to a moderate misclassification of frailty [56] and to a potential underestimation of its occurrence over time because of attrition. Moreover, the scarce availability of literature adopting a multistate approach, as well as the heterogeneity of criteria and methods used to evaluate frailty transitions, limits the comparability of our results with previously published findings. The major strengths of our study are its longitudinal design, the large population-based representative sample, the multistate modelling, and the availability of a comprehensive and reliable set of sociodemographic, clinical and subclinical information, assessed through standardized and well-established criteria by trained operators.

## 5 Conclusion

This study confirms the fluctuating course of frailty, offering useful information to orient time and characteristics of preventive measures aimed to maximise functional reserve and react to stressors. It provides evidence of an ample potential for remission/improvement over time, but also shows that women have a higher chance of worsening (and probable resilience), while men of death. Interventions at short/medium term should have the higher likelihood of being successful. Socio-psychological factors seem to play a determinant role in the development of frailty, highlighting the importance of a personalized, integrated approach. Further analysis of clusters of individuals with similar characteristics and longitudinal trajectories will be essential for risk stratification and prioritization of interventions.

## Funding

The Italian Longitudinal Study on Aging (ILSA) was supported, as part of the ‘Targeted Project on Aging’, by the Italian National Research Council (CNR) with grants to each research unit from 1991 to 1995. It was then funded by the Italian Ministry of Health (D.L. 502/92, 1998) through the programs ‘Epidemiology of the Elderly’ (Istituto Superiore di Sanità) and ‘Estimates of Health Needs of the Elderly’ (special programme of the Tuscany Region). The work conducted at ISS for the present analysis was supported by current research funding (Ricerca Corrente) from the Italian Ministry of Health.

Data availability statement

The complete dataset that support the findings of this study is not publicly available due to the original agreements regulating ILSA conduction and data management. Any further request of data not included in this article and/or in its online supplementary files can be directed to the corresponding author.

## Declaration of generative AI and AI-assisted technologies in the writing process

During the preparation of this work no generative AI tool or service has been used by the authors.

## CRediT authorship contribution statement

**Lucia Galluzzo:** Writing – original draft, Visualization, Methodology, Data curation, Conceptualization. **Marianna Noale:** Writing – original draft, Visualization, Methodology, Formal analysis, Conceptualization. **Stefania Maggi:** Writing – review & editing, Methodology, Funding acquisition. **Marzia Baldereschi:** Writing – review & editing, Project administration, Investigation. **Antonio Di Carlo:** Writing – review & editing, Project administration, Investigation. **Nicola Veronese:** Writing – review & editing, Validation. **Marco Silano:** Writing – review & editing, Supervision, Funding acquisition.

## Declaration of competing interest

The authors have no conflicts of interest to declare.

## Appendix Supplementary materials

Supplementary materials attached file FrailtyTransitionsILSA_SupplMaterials.docx Image, application 1
