# Supplementary material for: Transitions in frailty states and associated factors: a multistate analysis of the Italian Longitudinal Study on Aging population-based cohort
Source: J Frailty Aging. 2026 Jan 16;15(1):100117. doi: 10.1016/j.tjfa.2025.100117 (PMC12856321; doi:10.1016/j.tjfa.2025.100117)

**Supplementary Materials**

**Transitions in frailty states and associated factors: a multistate analysis of the Italian Longitudinal Study on Aging population-based cohort**

Lucia Galluzzo, Marianna Noale, Stefania Maggi, Marzia Baldereschi, Antonio Di Carlo, Nicola Veronese, Marco Silano, and the ILSA Working Group

**Supplementary Table 1.** Frailty transitions type, including death, in the ILSA cohort during each follow-up interval, by sex

**Supplementary Table 2.** Factors influencing transitions from frailty states in both sexes according to the multistate model adjusting for all covariates

**Supplementary Figure 1.** Underlying multistate model for examining frailty states transitions among ILSA study participants from T0 to T1, and from T1 to T2

**Supplementary Figure 2**. Transition intensity matrix based on the 4-state model and assuming a time-homogeneous Markov process

**Supplementary Figure 3.** Sex- and age-adjusted estimated survival probability for each frailty state, in whole participants and by sex

**Supplementary Table 1.** Frailty transitions type, including death, in the ILSA cohort during each follow-up interval, by sex

|  | T0-T1 | | | | | | T1-T2 | | | | | |
| --- | --- | --- | --- | --- | --- | --- | --- | --- | --- | --- | --- | --- |
|  | Women  (n=589) | | Men  (n=651) | | Whole  (n=1240) | | Women  (n=412) | | Men  (n=520) | | Whole  (n=932) | |
|  | n | % | n | % | n | % | n | % | n | % | n | % |
| Stable | 328 | 100.0% | 377 | 100.0% | 705 | 100.0% | 185 | 100.0% | 237 | 100.0% | 422 | 100.0% |
| Non-frail | 123 | 37.5% | 265 | 70.3% | 388 | 55.0% | 49 | 26.5% | 150 | 63.3% | 199 | 47.2% |
| Pre-frail | 194 | 59.1% | 109 | 28.9% | 303 | 43.0% | 123 | 66.5% | 79 | 33.3% | 202 | 47.9% |
| Frail | 11 | 3.4% | 3 | 0.8% | 14 | 2.0% | 13 | 7.0% | 8 | 3.4% | 21 | 5.0% |
| Worsened | 136 | 100.0% | 139 | 100.0% | 275 | 100.0% | 135 | 100.0% | 155 | 100.0% | 290 | 100.0% |
| Non-frail to pre-frail | 108 | 79.4% | 119 | 85.6% | 227 | 82.5% | 67 | 49.6% | 105 | 67.7% | 172 | 59.3% |
| Non-frail to frail | 2 | 1.5% | 5 | 3.6% | 7 | 2.5% | 10 | 7.4% | 11 | 7.1% | 21 | 7.2% |
| Pre-frail to frail | 26 | 19.1% | 15 | 10.8% | 41 | 14.9% | 58 | 43.0% | 39 | 25.2% | 97 | 33.4% |
| Improved | 72 | 100.0% | 62 | 100.0% | 134 | 100.0% | 44 | 100.0% | 61 | 100.0% | 105 | 100.0% |
| Pre-frail to non-frail | 54 | 75.0% | 57 | 91.9% | 111 | 82.8% | 35 | 79.5% | 55 | 90.2% | 90 | 85.7% |
| Frail to pre-frail | 17 | 23.6% | 5 | 8.1% | 22 | 16.4% | 9 | 20.5% | 6 | 9.8% | 15 | 14.3% |
| Frail to non-frail | 1 | 1.4% | 0 | 0.0% | 1 | 0.7% | 0 | 0.0% | 0 | 0.0% | 0 | 0.0% |
| Dead | 53 | 100.0% | 73 | 100.0% | 126 | 100.0% | 48 | 100.0% | 67 | 100.0% | 115 | 100.0% |
| Non-frail | 13 | 24.5% | 34 | 46.6% | 47 | 37.3% | 19 | 39.6% | 36 | 53.7% | 55 | 47.8% |
| Pre-frail | 31 | 58.5% | 32 | 43.8% | 63 | 50.0% | 23 | 47.9% | 24 | 35.8% | 47 | 40.9% |
| Frail | 9 | 17.0% | 7 | 9.6% | 16 | 12.7% | 6 | 12.5% | 7 | 10.4% | 13 | 11.3% |

**Supplementary Table 2.** Factors influencing transitions from frailty states in both sexes according to the multistate model adjusting for all covariates

| Covariates | Transitions | | | | | | | | |
| --- | --- | --- | --- | --- | --- | --- | --- | --- | --- |
|  | Non-frail to | | | Pre-frail to | | | Frail to | | |
|  | Pre-frail | Frail | Dead | Non-frail | Frail | Dead | Non-frail | Pre-frail | Dead |
| Sex,  females | **1.31**  **(1.02-1.67)** | 1.13  (0.53-2.43) | 1.16  (0.78-1.75) | **0.63**  **(0.45-0.88)** | 0.86  (0.57-1.28) | 0.97  (0.66-1.42) | 34.49  (0-274149.2) | 0.84  (0.30-2.36) | 0.27  (0.07-1.11) |
| Age,  years | 1.01  (0.99-1.03) | **1.08**  **(1.02-1.15)** | **1.09**  **(1.06-1.12)** | **0.94**  **(0.92-0.97)** | **1.08**  **(1.04-1.11)** | **1.06**  **(1.03-1.1)** | 1.01  (0.66-1.55) | 0.98  (0.91-1.06) | 1.06  (0.94-1.2) |
| Schooling,  <8 years | 0.97  (0.78-1.21) | 0.94  (0.46-1.93) | 0.95  (0.66-1.34) | 0.9  (0.66-1.24) | 1.11  (0.72-1.71) | 0.99  (0.69-1.43) | 11.11  (0-289913.6) | 1.25  (0.46-3.41) | 0.46  (0.13-1.6) |
| Marital status, not married | 1.01  (0.76-1.36) | 1.15  (0.53-2.46) | 0.93  (0.57-1.54) | 0.76  (0.51-1.15) | 0.93  (0.63-1.39) | 1.37  (0.93-2.02) | 0.87  (0.01-80.98) | 1.29  (0.53-3.14) | 1.04  (0.27-3.99) |
| Liv. arrangement, living alone | 0.94  (0.66-1.33) | 0.39  (0.13-1.23) | 1.52  (0.88-2.62) | 1.14  (0.68-1.89) | 0.91  (0.56-1.47) | 1.36  (0.90-2.05) | - | 0.89  (0.27-2.92) | 0.76  (0.07-8.33) |
| Smoking habit, smoker/ex | 1.08  (0.82-1.43) | 0.96  (0.45-2.05) | 1.14  (0.72-1.82) | 1.14  (0.76-1.7) | 0.68  (0.47-1.00-) | **1.6**  **(1.01-2.51)** | 0.13  (0-11.58) | 0.48  (0.23-1.01) | 0.97  (0.28-3.34) |
| Alcohol habit, drinker/ex | 1  (0.80-1.25) | 0.65  (0.30-1.39) | **1.51**  **(1.06-2.15)** | 0.97  (0.70-1.34) | 0.76  (0.49-1.17) | 0.76  (0.49-1.17) | 1.82  (0.28-2.59) | 0.03  (0-718.18) | 0.78  (0.30-2.03) |
| BMI, underweight /obese | 1.09  (0.85-1.39) | 1.37  (0.68-2.76) | 1.1  (0.73-1.65) | 1.1  (0.73-1.65) | 0.71  (0.49-1.03) | **1.63**  **(1.15-2.31)** | 1.04  (0.72-1.51) | 0.02  (0-3325.05) | 1.71  (0.78-3.76) |
| Comorbidity,  ≥2 | 1.12  (0.91-1.37) | 1.38  (0.75-2.54) | 0.95  (0.68-1.32) | **0.54**  **(0.39-0.74)** | 1.35  (0.97-1.86) | **1.97**  **(1.44-2.69)** | 0.11  (0-20.06) | 1.04  (0.51-2.09) | 1.23  (0.49-3.05) |
| Cognitive impair., MMSE <24 | 1.21  (0.83-1.75) | 0.92  (0.32-2.66) | 1.08  (0.59-1.96) | 1.17  (0.71-1.92) | 1.38  (0.90-2.09) | 1.1  (0.70-1.73) | 0.02  (0-2018.12) | 0.50  (0.18-1.40) | 1.73  (0.56-5.33) |
| Depressive sympt., GDS ≥10 | 1.09  (0.85-1.40) | **3.7**  **(1.94-7.05)** | 1.25  (0.85-1.85) | **0.7**  **(0.50-0.98)** | **1.46**  **(1.05-2.05)** | 1.24  (0.90-1.71) | - | 0.61  (0.23-1.61) | 2.97  (0.27-33.16) |
| Falls,  ≥ 1 | 0.9  (0.7-1.15) | 1.41  (0.73-2.74) | **0.62**  **(0.4-0.96)** | 1.07  (0.77-1.49) | 1.29  (0.92-1.82) | 0.77  (0.54-1.11) | 0.03  (0-3884.31) | 0.88  (0.41-1.89) | 1.1  (0.40-3.04) |

Values are expressed as Hazard Ratios (95% Confidence Intervals), using as reference the complementary category/ies at lower frailty risk from bivariate analysis.

Statistically significant association (CI not including 1.00) are presented in bold.

**Supplementary Figure 1.** Underlying multistate model for examining frailty states transitions among ILSA study participants from T0 to T1, and from T1 to T2

λ_22_

λ_11_

λ_21_

λ_12_

1. Non-frail

2. Pre-frail

λ_24_

λ_14_

4. Death

λ_23_

λ_32_

λ_13_

λ_31_

λ_34_

3. Frail

λ_33_

**Supplementary Figure 2.** Transition intensity matrix based on the 4-state model and assuming a time-homogeneous Markov process

| Λ = |  | - (λ_12_ + λ_13_ + λ_14_) | λ_12_ | λ_13_ | λ_14_ |  |
| --- | --- | --- | --- | --- | --- | --- |
|  |  | λ_21_ | - (λ_21_ + λ_23_ + λ_24_) | λ_23_ | λ_24_ |  |
|  |  | λ_31_ | λ_32_ | - (λ_31_ + λ_32_ + λ_34_) | λ_34_ |  |
|  |  | 0 | 0 | 0 | 0 |  |

**Supplementary Figure 3.** Sex- and age-adjusted estimated survival probability for each frailty state, in whole participants and by sex


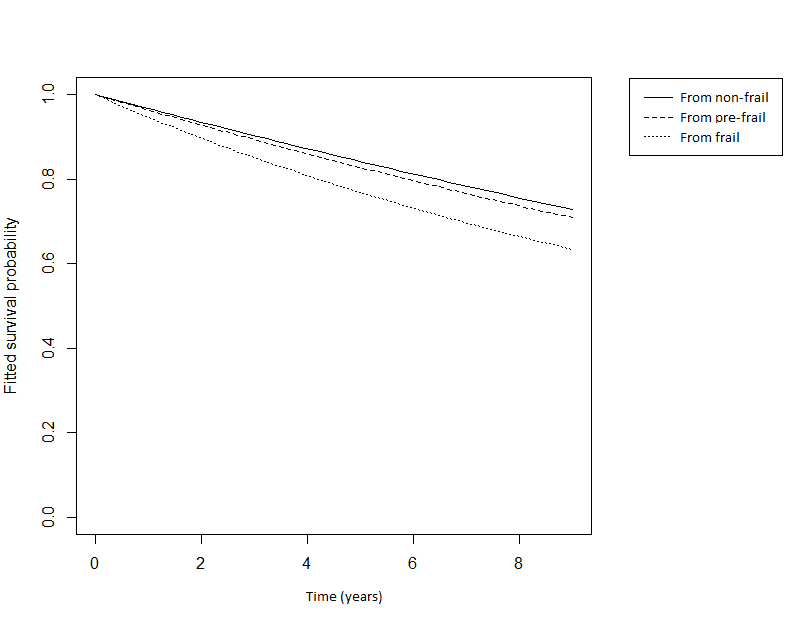


Overall

Men

Women


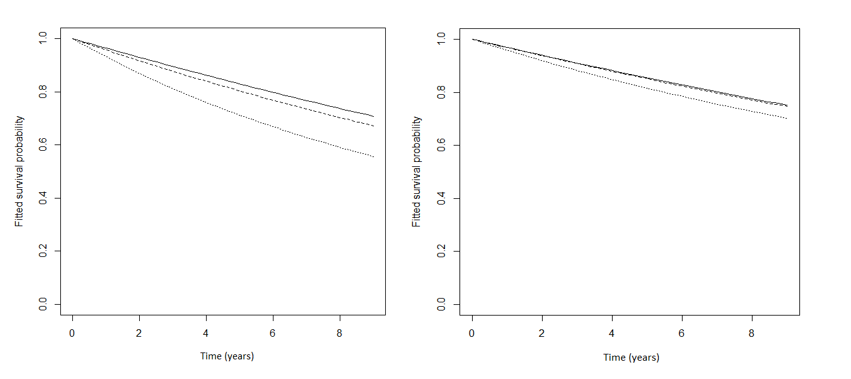

Supplement: Supplementary file 1 — Supplementary materials attached file FrailtyTransitionsILSA_SupplMaterials.docx [file mmc1.docx]
